# Prognostic value of pre-operative peripheral inflammation markers in patients with squamous cell carcinoma of the external auditory canal

**DOI:** 10.1016/j.bjorl.2020.05.018

**Published:** 2020-06-24

**Authors:** Feitian Li, Xunbei Shi, Chunfu Dai

**Affiliations:** a Fudan University, Eye, Ear, Nose and Throat Hospital, Department of Otology and Skull Base Surgery, Shanghai, People’s Republic of China; b Key Laboratory of Hearing Medicine, Ministry of Health, Eye, Ear, Nose, and Throat Hospital, Fudan University, Shanghai, People’s Republic of China

**Keywords:** Ear, Cancer, Lymphocyte, Neutrophil, Prognostic factors

## Abstract

**Introduction:**

Squamous cell carcinoma of the external auditory canal is a rare entity. Previous studies have suggested predictors for tumor recurrence. However, most of the prognostic factors were from the clinicopathological aspect.

**Objective:**

This study aims to analyze the correlation between pre-operative peripheral inflammation markers and survival outcomes, in order to identify prognostic biomarkers for patients with squamous cell carcinoma of the external auditory canal.

**Methods:**

We retrospectively analyzed patients diagnosed with squamous cell carcinoma of the external auditory canal who underwent surgery at our institute. The pre-operative circulating inflammatory markers, such as the neutrophil, lymphocyte, platelet, and monocyte counts were measured and their ratios including neutrophil-to-lymphocyte ratio, platelet-to-lymphocyte ratio and lymphocyte-to-monocyte ratio were calculated. The prognostic value of the measured hematologic parameters in relation to the survival outcomes was also evaluated.

**Results:**

A total of 83 patients were included, of which 26 patients showed tumor recurrence and 57 without recurrence. Neutrophil counts and neutrophil-to-lymphocyte ratio were closely connected with tumor stage. In the patients with recurrence, neutrophil counts, neutrophil-to-lymphocyte ratio and platelet-to-lymphocyte ratio were elevated (*p* < 0.0001, *p* < 0.0001 and *p* = 0.001), while lymphocyte counts and lymphocyte-to-monocyte ratio were decreased (*p* = 0.013 and *p* = 0.016, respectively). The receiver operating curve analysis indicated that pre-operative neutrophil-to-lymphocyte ratio is a potential prognostic marker for recurrence of squamous cell carcinoma of the external auditory canal (area under curve = 0.816), and the cut-off points was 2.325.

**Conclusions:**

Pre-operative neutrophil and lymphocyte counts, neutrophil-to-lymphocyte ratio, platelet-to-lymphocyte ratio, lymphocyte-to-monocyte are significantly correlated with tumor recurrence in patients with external auditory canal squamous cell carcinoma. Furthermore, neutrophil-to-lymphocyte ratio may be unfavorable prognostic factors of this disease.

## Introduction

Squamous cell carcinoma (SCC) of the external auditory canal is a rare malignancy. Thus, limited data are available for this cancer. The current treatment strategy for external auditory canal SCC is either surgery, radiotherapy, chemotherapy, or a combination of these. At the moment, there is no staging system approved by the Union for International Cancer Control or by the American Joint Committee on Cancer for this type of malignancy. The Pittsburgh staging system, which was first proposed in 1996 and modified in 2000, is commonly used in clinical practice to select the surgical approach and to evaluate the survival outcome.^1^, ^2^ Most studies on external auditory canal SCC only analyzed well-established prognostic factors, including the tumor stage, involved structures, and combined adjuvant therapy. However, by only focusing on local invasion, other information, such as tumor immunology and cellular biology may be overlooked. Therefore, new prognostic factors need to be identified.

Since Virchow first described the presence of leukocytes in neoplastic tissues and the correlation of inflammation with cancer in 1863, numerous studies have attempted to elucidate the connection between inflammation and malignancy progression.^3^, ^4^, ^5^ In recent years, mounting evidence has suggested that counts of different cell types in the circulating blood are associated with survival rates in various malignancies, including gastric cancer, oral carcinoma and nasopharyngeal carcinoma. Additionally, the hematologic ratios, such as the Neutrophil-to-Lymphocyte Ratio (NLR), Platelet-to-Lymphocyte Ratio (PLR), and Lymphocyte-to-Monocyte Ratio (LMR), were found to predict the prognosis of patients affected by various cancers. The role of inflammatory cells in the cancer micro-environment and metastasis is also emerging in laboratory research. Lartigue et al.^6^ found that circulating tumor cells could be trapped within neutrophil-derived DNA webs after systemic infection, implicating that neutrophils are involved in tumor metastasis. Labelle et al.^7^ demonstrated that, after release of TGF-β and direct contact with tumor cells, platelets can activate tumor TGFβ/Smad and NF-κB pathways, enhancing tumor transition toward a more invasive phenotype and metastasis. Reichert et al.^8^ showed that the tumor-mediated death of circulating T-cells through the Fas/Fas L pathway suppressed the immune anti-tumoral function in oral carcinoma. These findings provide an in-depth understanding of the involvement of inflammatory markers in carcinogenesis, which could provide an evidence base for development of new strategies to manage cancer patients. However, to our knowledge, the prognostic value of pre-operative inflammatory markers in external auditory canal SCC has not yet been tested.

Therefore, in this study, we evaluated the correlation between pre-operative inflammatory markers and prognosis in patients affected with external auditory canal SCC in order to identify new independent prognostic factors for this rare disorder.

## Methods

### Study population

This study was approved by the medical ethics committee of the Eye, Ear, Nose and Throat Hospital of Fudan University (2014007), and all patients signed informed consent forms.

We performed a retrospective review of all patients diagnosed with external auditory canal SCC who underwent surgery at our institution between January 2005 and December 2018. The inclusion criteria were the following: 1) Primary external auditory canal SCC diagnosis confirmed by a histopathological examination at our institution; 2) Curative temporal bone resection with parotidectomy was performed; 3) Consent for enrollment in the study; 4) Comprehensive medical records, including imaging, laboratory test, and follow-up were available. The following patients were excluded: 1) Patients who underwent pre-operative radiotherapy or chemotherapy; 2) Patients who had a concurrent hematologic disease, systemic inflammatory disease, or infection; 3) Patients who were diagnosed with auto-immune disease or were treated with immune-suppressive agents; 4) Patients who were affected with a suspected distant metastasis after computed tomography and magnetic resonance imaging. Ninety-eight patients met the inclusion criteria, of which 15 patients were later excluded from the survival analysis due to a loss to follow-up.

### Data collection

We collected medical records for all the patients involved in the study included laboratory tests results, and radiological and histological findings. Patients were staged according to the Pittsburgh staging system. The patients’ blood samples were collected in ethylenediaminetetraacetic acid-containing tubes within 2 weeks before the operation. Pre-operative neutrophil, monocyte, lymphocyte, and platelet counts were measured using the Mindary BC-5500 (Shenzhen, China) automatic blood counting system. The NLR, PLR, and LMR were calculated by dividing the absolute values of the corresponding cell counts.

### Patient follow-up

All patients were followed up with interval MRI and chest computed tomography after surgery to detect local recurrence or distant metastasis. The last follow-up for all patients was in October 2019. The follow-up duration ranged from 8 to 138 months with a median of 27 months.

### Statistical analysis

The Mann-Whitney *U* test and Kruskal Wallis test were used to compare the mean pre-operative hematologic parameters and calculated ratios stratified by demographics and clinical characteristics. Mann-Whitney *U* test was applied to compare the pre-operative hematologic parameters and calculated ratios between recurs and non-recur patients. Data are presented as mean ± SD. The Receiver-Operating Curve (ROC) analysis was performed to acquire the Youden index (sensitivity + specificity-1), and the optimal cut-off points of neutrophil, monocyte, lymphocyte, platelet counts and NLR, PLR, LMR were calculated. Results were considered significant if two-sided was *p* < 0.05. All statistical analyses were conducted using the IBM SPSS 20.0 software (IBM, Armonk, NY, USA).

## Results

### Demographics and clinical characteristics

The patients’ demographics and clinical characteristics are shown in Table 1. The patient cohort was composed of 59 males and 24 females (male: female ratio = 2.46:1) with a mean age of 60.78 ± 9.24 years (range, 35–79 years). The median follow-up time was 27 months (range, 8–138 months). According to the Pittsburgh staging system, 24 patients (28.9%) were at an early stage (T1–T2), whereas 59 patients (71.1%) were at an advanced stage (T3–T4). Furthermore, 4 patients (4.8%) had lymph node metastasis. In 42 patients (50.6%), the tumors were well or moderately differentiated, in 7 (8.4%), they were poorly differentiated, and in 34 patients (41.0%), this was unknown.

**Table 1:** Patients characteristics.

Clinical characteristics	Numbers
Age (< 60 years/ ≥ 60 years)	33 (39.8%)/50 (60.2%)
Sex (male/female)	59 (71.1%)/24 (28.9%)
Stage	
T1	3 (3.6%)
T2	21 (25.3%)
T3	22 (26.5%)
T4	37 (44.6%)
Lymph node status	
N0	79 (95.2%)
N1	4 (4.8%)
Differentiation patterns	
Well & moderately differentiated	42 (50.6%)
Poorly differentiated	7 (8.4%)
Unknown	34 (41.0%)

### Correlation of hematologic parameters with demographic and clinicopathological characteristics

Comparison of hematological and clinicopathological parameters are shown in Table 2, Table 3. Since lymph node metastasis only occurred in 4 patients, we did not further analyze the correlation between lymph node metastasis and hematologic parameters, as no significant correlation could be drawn in this cohort.

**Table 2:** Means of pre-operative hematologic parameters (×10^9^), stratified by demographic and clinicopathological characteristics.

Variables	Neu	*p*	Mon	*p*	Lym	*p*	Plt	*p*
	(Mean ± SD)		(Mean ± SD)		(Mean ± SD)		(Mean ± SD)	
Age		0.105		0.180		0.186		0.058
<60 years	3.91 ± 1.02		0.47 ± 0.16		1.72 ± 0.69		209.20 ± 51.68	
≥ 60 years	3.66 ± 1.35		0.42 ± 0.16		1.57 ± 0.60		193.60 ± 61.05	
Sex		0.077		0.074		0.599		0.357
M	3.34 ± 0.82		0.39 ± 0.11		1.64 ± 0.63		205.10 ± 50.54	
F	3.93 ± 1.33		0.46 ± 0.17		1.70 ± 0.61		197.30 ± 60.60	
Stage		**0.018**		0.226		0.597		0.981
T1–T2	3.26 ± 0.81		0.40 ± 0.13		1.73 ± 0.68		201.80 ± 63.00	
T3	3.66 ± 1.23		0.45 ± 0.20		1.80 ± 0.78		196.40 ± 50.21	
T4	4.15 ± 1.34		0.46 ± 0.14		1.58 ± 0.42		200.60 ± 59.72	
Differentiation pattern		0.074		0.981		0.291		0.770
Well & moderate	4.33 ± 1.49		0.47 ± 0.15		1.74 ± 0.65		208.20 ± 54.49	
Poor	3.54 ± 1.37		0.48 ± 0.23		1.54 ± 0.71		200.00 ± 74.78	

Neu, neutrophils; Mon, monocytes; Lym, lymphocytes; Plt, platelets.

**Table 3:** Means of pre-operative NLR, PLR, and LMR, stratified by demographic and clinicopathological characteristics.

Variables	NLR	*p*	PLR	*p*	LMR	*p*
	(Mean ± SD)		(Mean ± SD)		(Mean ± SD)	
Age		0.474		0.647		0.665
< 60 years	2.50 ± 1.50		130.20 ± 59.88		4.22 ± 1.74	
≥ 60 years	2.59 ± 1.64		134.40 ± 68.38		4.17 ± 1.92	
Sex		0.400		0.216		0.132
M	2.30 ± 1.24		137.60 ± 51.71		4.61 ± 1.92	
F	2.66 ± 1.69		130.70 ± 69.72		4.02 ± 1.80	
Stage		0.009		0.665		0.212
T1–T2	2.04 ± 0.61		124.60 ± 38.05		4.56 ± 1.84	
T3	2.62 ± 2.33		130.60 ± 73.00		4.42 ± 2.10	
T4	2.85 ± 1.41		139.20 ± 73.74		3.82 ± 1.65	
Differentiation pattern		0.542		0.962		0.218
Well & moderate	2.90 ± 1.80		140.00 ± 78.67		4.01 ± 1.65	
Poor	2.67 ± 1.30		173.90 ± 155.20		3.26 ± 1.20	

M, male; F, female; NLR, neutrophil-to-lymphocyte ratio; PLR, platelet-to-lymphocyte ratio; LMR, lymphocyte-to-monocyte ratio.

We found no significant differences between the hematologic parameters and demographic characteristics, such as age and sex. However, we found that neutrophil counts and NLR are significantly associated with the tumor stage. Patients with advanced stage cancer (T3–4) had higher neutrophil counts and NLR than those in the early stage (T1–2). In addition we found no significant correlation between the differentiation patterns and any hematologic parameters.

### Correlation of hematologic parameters with follow-up outcomes

During follow-up, we observed 26 recurrences. Of the patients showing recurrence, 18 eventually died of EACSCC, while 8 were alive with local recurrence at their last follow-up. Fifty-seven patients showed no recurrence, among them 55 patients were alive without evidence of disease, while two died of cardiac disease and colon cancer, respectively. None of the 83 patients developed distant metastasis.

**Figure 1:**
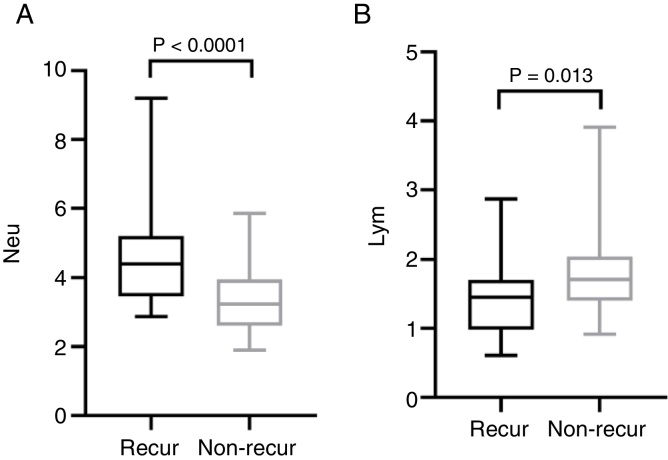
Comparison of neutrophil (A), lymphocyte (B) counts in recur and non-recur patients.

**Figure 2:**
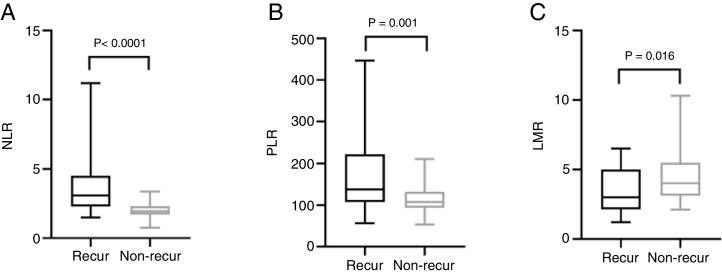
Comparison of NLR (A), PLR (B) and LMR (C) in recur and non-recur patients. NLR, neutrophil-to-lymphocyte ratio; PLR, platelet-to-lymphocyte ratio; LMR, lymphocyte-to-monocyte ratio.

The mean pre-operative neutrophil, monocyte, lymphocyte and platelet counts was 4.56 ± 1.47, 0.46 ± 0.15, 1.44 ± 0.57 and 212.50 ± 62.10 in recur patients, and 3.40 ± 0.91, 0.43 ± 0.16, 1.79 ± 0.60, 194.0 ± 55.18 in non-recur patients, respectively. The mean pre-operative NLR, PLR, LMR was 3.76 ± 2.70, 174.20 ± 93.92, 3.50 ± 1.69 in recur patients and 2.00 ± 0.55, 113.80 ± 32.31, 4.50 ± 1.84 in non-recur patients, respectively. Mann-Whitney test revealed that neutrophil counts, NLR and PLR significantly increased in recur patients (*p* <  0.0001, *p* <  0.0001 and *p* =  0.001, respectively), while lymphocyte counts and LMR decreased in recur patients (*p* =  0.013 and *p* =  0.016, respectively) (Figure 1, Figure 2).

### Prognostic markers for EACSCC recurrence

**Figure 3:**
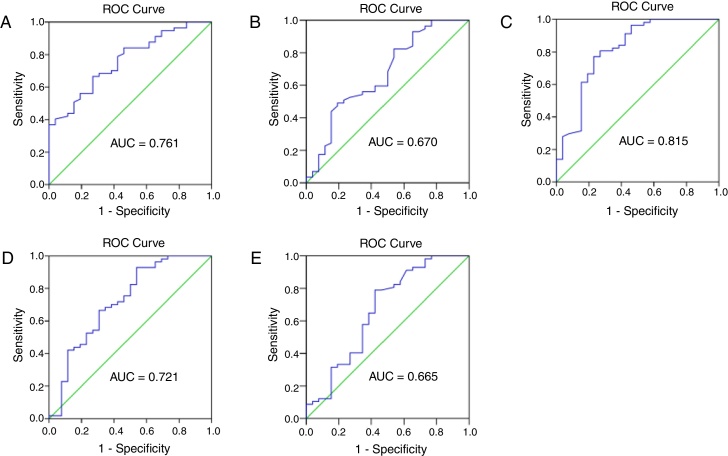
The receiver operating characteristic (ROC) curve and area under curve (AUC) to analyze the optimal cut-off points for neutrophil (A) and lymphocyte (B) counts, NLR (C), PLR (D) and LMR (E). NLR, neutrophil-to-lymphocyte ratio; PLR, platelet-to-lymphocyte ratio; LMR, lymphocyte-to-monocyte ratio.

The prognostic ability of hematological parameters and ratios for patients with external auditory canal SCC were analyzed by the ROC analyses. The results are shown in Fig. 3. The Area Under Curve (AUC) of neutrophil, lymphocyte, platelet and monocyte counts was 0.761, 0.670, 0.618 and 0.552, with the 95% Confidence Interval varying from 0.657 to 0.866, 0.539‒0.801, 0.495‒0.741 and 0.422‒0.482, and *p* <  0.001, <0.05, <0.05 and <0.05, respectively. The AUC of NLR, PLR and LMR was 0.815, 0.721, 0.665, with the 95% Confidence Interval varying from 0.707 to 0.924, 0.593‒0.848, 0.528‒0.803, and *p* < 0.0001, <0.01, <0.05, respectively. The cut-off points were calculated between recur and non-recur patients. The optimal cut-off points were 3.75×10^9^/L for neutrophil counts, 1.77 × 10^9^/L for lymphocyte counts, 2.325 for NLR, 157.9 for PLR and 3.065 for LMR.

Our study revealed that in patients with external auditory canal SCC, pre-operative neutrophil and lymphocyte counts, NLR, PLR and LMR were associated with tumor recurrence. NLR may be unfavorable prognostic factors for survival outcome of EACSCC patients.

## Discussion

This study evaluated the prognostic value of comprehensive pre-operative inflammatory hematologic parameters (neutrophil, platelet, lymphocyte, and monocyte counts) and calculated ratios (NLR, PLR and LMR) for patients with external auditory canal SCC. Our results show that pre-operatively elevated neutrophil counts, NLR, PLR and decreased lymphocyte counts, LMR were significantly correlated with recurrence of EACSCC. In terms of tumor progression, our results showed that neutrophil counts and NLR were correlated with tumor stage. Although the prognostic value of pre-operative hematologic parameters had already been validated for various tumors, including head and neck cancers, our study was the first to evaluate their prognostic value for external auditory canal SCC patients.

Previous studies have reported that males have higher neutrophil, lymphocyte, and platelet counts than females.^9^, ^10^ However, in this study we observed no statistical difference in the blood cells counts and ratios between the sexes and ages. Our study showed that an increase in neutrophil counts and NLR are correlated with the tumor stage. Neutrophils are bone marrow-derived myeloid cells that can be recruited to tumors, where they promote tumor progression by facilitating angiogenesis, inhibiting T-cell activity, and participating in other biological events.^11^, ^12^ This may explain the correlation between tumor stage and neutrophil counts, and NLR.

What is more, neutrophils have an established dual role in the host’s defense toward infection and secreting cytokines and enzymes that stimulate tumor growth, promoting tumor angiogenesis and metastasis.^13^ Additionally, neutrophils can behave as immunosuppressive cells by inhibiting T-cell activity through the production of nitrogen and reactive oxygen species.^14^ This observation can explain why NLR is a useful prognostic factor for cancer patients. Balermpas et al.^15^ reported that a favorable survival rate in patients with head and neck cancer was associated with higher pre-treatment levels of infiltrating CD3+ and CD8+ lymphocytes. Zhang et al.^16^ showed that the absence of intra-tumoral T-cells in ovarian carcinoma is associated with an increased expression of the Vascular Endothelial Growth Factor (VEGF), which promotes tumor angiogenesis. The presence of intra-tumoral T-cells is correlated with a late recurrence and an improved survival rate. Platelets can stimulate tumor metastasis by promoting cell aggregation to shield tumor cells from immune surveillance. Increased platelet levels can stimulate osteoclast activity and facilitate bone metastasis.^17^ Circulating monocytes are recruited to the tumor site, where they differentiate into Tumor-Associated Macrophages (TAMs) by tumor-produced chemokines. TAMs produce a variety of molecules that promotes tumor angiogenesis and stimulates tumor cell growth, such as VEGF, IL-1, hypoxia-inducible factor-2α, fibroblast growth factor and hepatocyte growth factor. TAMs also contribute to tumor metastasis by producing basement membrane proteases.^18^ In summary, extensive studies have demonstrated interactions between peripheral hematologic parameters and tumors.

Previous studies reported that NLR significantly predicts a worse outcome in various cancers.^19^, ^20^ And high neutrophil and monocyte circulating levels, and low circulating lymphocyte levels were correlated with poor prognosis.^21^, ^22^ Furthermore, Rachidi et al.^23^ independently confirmed that a lower platelet count predicted a better prognosis, and that administration of anti-platelet agents improved survival of patients with head and neck SCC. Xie et al.^24^ showed that pre-operative PLR inversely correlated with Cancer Specific Survival (CSS) in patients with esophageal SCC. Several studies have reported that the LMR is associated with prognosis in cancer patients. These results validate the use of peripheral inflammation markers to predict postoperative outcomes in cancer patients. In this study, we found the pre-operative neutrophil counts, NLR, PLR were higher, while lymphocyte counts and LMR were lower in patients with recurrence compare to the non-recur patients. As shown in Fig. 3, the cut-off points were 3.75 × 10^9^/L for neutrophil counts, 1.77 × 10^9^/L for lymphocyte counts, 2.325 for NLR, 157.9 for PLR and 3.065 for LMR in our study. NLR can serve as a potential prognostic marker in clinical practice with an ideal AUC value of 0.816.

However, this study had some limitations, including its retrospective design and the relatively small sample size, which may lead to referral biases. Therefore, further studies are necessary to validate the role and the prognostic utility of systemic inflammatory markers in such patients.

## Conclusions

Taken together, our results suggested that pre-operative neutrophil and lymphocyte counts, NLR, PLR, and LMR are significantly in relation to EACSCC recurrence. Furthermore, NLR may serve as potential prognostic markers to predict worse outcomes in patients with external auditory canal SCC.

## Funding

This study was funded by the 10.13039/501100001809National Natural Science Foundation of China (nº 81771009).

## Conflicts of interest

The authors declare no conflicts of interest.
